# Identification of a Novel Transcription Factor TP05746 Involved in Regulating the Production of Plant-Biomass-Degrading Enzymes in *Talaromyces pinophilus*

**DOI:** 10.3389/fmicb.2019.02875

**Published:** 2019-12-13

**Authors:** Ting Zhang, Lu-Sheng Liao, Cheng-Xi Li, Gui-Yan Liao, Xiong Lin, Xue-Mei Luo, Shuai Zhao, Jia-Xun Feng

**Affiliations:** State Key Laboratory for Conservation and Utilization of Subtropical Agro-Bioresources, Guangxi Research Center for Microbial and Enzyme Engineering Technology, College of Life Science and Technology, Guangxi University, Nanning, China

**Keywords:** transcription factor, plant-biomass-degrading enzymes, mycelial development, conidiation, *Talaromyces pinophilus*

## Abstract

Limited information on transcription factor (TF)-mediated regulation exists for most filamentous fungi, specifically for regulation of the production of plant-biomass-degrading enzymes (PBDEs). The filamentous fungus, *Talaromyces pinophilus*, can secrete integrative cellulolytic and amylolytic enzymes, suggesting a promising application in biotechnology. In the present study, the regulatory roles of a Zn2Cys6 protein, TP05746, were investigated in *T. pinophilus* through the use of biochemical, microbiological and omics techniques. Deletion of the gene *TP05746* in *T. pinophilus* led to a 149.6% increase in soluble-starch-degrading enzyme (SSDE) production at day one of soluble starch induction but an approximately 30% decrease at days 2 to 4 compared with the parental strain Δ*TpKu70*. In contrast, the *T*. *pinophilus* mutant Δ*TP05746* exhibited a 136.8–240.0% increase in raw-starch-degrading enzyme (RSDE) production, as well as a 90.3 to 519.1% increase in cellulase and xylanase production following induction by culturing on wheat bran plus Avicel, relative to that exhibited by Δ*TpKu70*. Additionally, the mutant Δ*TP05746* exhibited accelerated mycelial growth at the early stage of cultivation and decreased conidiation. Transcriptomic profiling and real-time quantitative reverse transcription-PCR (RT-qPCR) analyses revealed that TP05746 dynamically regulated the expression of genes encoding major PBDEs and their regulatory genes, as well as fungal development-regulated genes. Furthermore, *in vitro* binding experiments confirmed that TP05746 bound to the promoter regions of the genes described above. These results will contribute to our understanding of the regulatory mechanism of PBDE genes and provide a promising target for genetic engineering for improved PBDE production in filamentous fungi.

## Introduction

Soil filamentous fungi play crucial roles in terrestrial carbon cycling because they can decay organic matter, including plant biomass (Gougoulias et al., 2014) via secretion of plant-biomass-degrading enzymes (PBDEs), such as cellulase, hemicellulose, and amylase (Bornscheuer et al., 2014). Most of the PBDEs are produced in nature by *Trichoderma*, *Penicillium* and *Aspergillus*, but they generate only low yields (Passos et al., 2018). Therefore, genetic improvement of natural fungal producers such as enzymes needs to be further explored, with an aim of developing strains capable of high production of PBDEs.

In the enzyme market, cellulases and amylases account for the majority of PBDEs. Cellulases are the complexes comprising cellobiohydrolases (EC 3.2.1.91; CBHs), endo-β-1,4-glucanases (EC 3.2.1.4; EGs), and β-1,4-glucosidases (EC 3.2.1.21; BGLs)^1^. Synergistic action among these enzymes is required for cellulose hydrolysis into glucose. During the process, EGs randomly break internal β-1,4-glycosidic bonds in glucan chains to release glucooligosaccharides, resulting in new chain ends. CBHs act on both ends of glucooligosaccharide chains to produce mainly cellobiose. Both soluble glucooligosaccharide and cellobiose are hydrolyzed by BGLs to yield glucose (Bornscheuer et al., 2014).

Comparably, amylases are classified broadly into glucoamylases (EC 3.2.1.3; GLAs), α-amylases (EC 3.2.1.1; AMYs), α-glucosidases (EC 3.2.1.20; AGAs) and 1,4-α-glucan-branching enzymes (EC 2.4.1.18) according to the action principle (see text footnote 1). Among them, GLAs attack α-1,4- or α-1,6-glucosidic bonds at the non-reducing ends of starch chains to release glucose, while AMYs break internal α-1,4-glycosidic bonds of starch chains to generate straight and branched oligosaccharides or maltose. AGAs digest maltose into glucose (Marín-Navarro and Polaina, 2011). Moreover, amylolytic enzymes can show activity toward soluble starch, known as soluble starch-degrading enzymes (SSDEs). Some amylolytic enzymes, known as raw starch-degrading enzymes (RSDEs), can directly hydrolyze raw starch granules below the starch gelatinization temperature (Sun et al., 2010). SSDEs are commonly used for conventional starch processing, including two-step liquefaction and saccharification. During liquefaction, raw starch is first gelatinized at a high temperature (95–105°C) and then liquefied into dextrin via the thermophilic AMYs. Subsequently, GLAs are applied in the saccharification step to hydrolyze the cooled dextrin into glucose at 60–65°C (Sánchez and Cardona, 2008; Görgens et al., 2015). However, the conventional liquefaction step requires high energy input and extra equipment, resulting in a high cost of the starch-derived products.

RSDEs contain the specific starch-binding domain (SBD) that enables them to bind onto the surface of raw starch granules (Sorimachi et al., 1997; Machovic and Janecek, 2006; Xu et al., 2016). RSDE application can reduce the process cost since the liquefaction step would not be needed (Sun et al., 2010; Lee et al., 2012). It was estimated that the application of RSDE in ethanol production, using starch as feedstock, could reduce the fuel cost of the total ethanol product by about 10–20% (Robertson et al., 2006).

Genetic engineering of fungal strains, based on the regulatory network of transcription factors (TFs) and their target genes, is an efficient strategy by which to improve functional enzyme yields. TFs are proteins that control transcription of target genes through binding to specific DNA sequence elements such as promoters, enhancers, etc. TF-mediated transcriptional control is a central regulatory mechanism in all eukaryotic organisms (Weirauch and Hughes, 2011). More than 90 types of TFs in eukaryotes have been identified, including zinc finger (Zn2Cys6, C2H2, GATA, CCHC, DHHC, etc.), helix-turn-helix (HTH), basic leucine zipper (bZIP), APSES and winged helix repressor DNA-binding domain (Weirauch and Hughes, 2011; Carrillo et al., 2017). The regulatory roles of individual TFs vary under different conditions.

Transcriptional expression of genes encoding PBDEs is strictly controlled by specific TFs in filamentous fungi. Since XlnR was first identified to be involved in regulating the expression of genes encoding enzymes involved in degrading plant cell walls in *Aspergillus* (van Peij et al., 1998), several TFs have been found to be expressed in the presence of recalcitrant carbon sources, such as the key activators CLR-2 (Coradetti et al., 2012; Zhao et al., 2016), AmyR (Li et al., 2015), and PoxCxrA (Yan et al., 2017). Among them, CLR-2 and PoxCxrA encode Gal4-like Zn2Cys6 proteins, and play essential roles in the regulation of fungal cellulase gene expression in the presence of cellulose. In *Neurospora crassa*, the expression of *clr-2* is induced by the TF CLR-1 (Coradetti et al., 2012), while it is induced by PoxCxrA in *Penicillium oxalicum* (Liao et al., 2019). The CLR-1 homolog ClrA is less involved in the regulation of cellulase gene expression in *P. oxalicum* (Liao et al., 2019), as well as in *Aspergillus nidulans* (Coradetti et al., 2012; Tani et al., 2014).

The Zn2Cys6 proteins AmyR and/or COL-26 are necessary for amylolytic gene expression in filamentous fungi such as *P. oxalicum*, *N. crassa*, *T. pinophilus*, *Aspergillus* spp., and are required for starch and maltose utilization (Li et al., 2015; Xiong et al., 2017; Zhang et al., 2017). Additionally, AmyR inhibits the expression of cellulase genes and its expression is regulated by *ClrB* in *P. oxalicum* (Li et al., 2015). COL-26 regulates cellulase gene expression and enzyme production, synergistically functioning with the carbon catabolite repressor, CRE-1 (Xiong et al., 2014).

CRE-1 mediates carbon catabolite repression (CCR) that represses cellulase gene expression in the presence of favorable carbon source such as glucose. CRE-1 could directly bind to the promoter regions of major cellulase and xylanase genes and their regulatory genes, such as *clr-2*, resulting in reduction of enzyme yields (Li et al., 2015; Huberman et al., 2016).

The soil fungus *T. pinophilus*, formerly called *Penicillium pinophilus*, produces useful PBDEs such as α-amylase, glucoamylase, cellulase, xylanase, and laccase (Pol et al., 2012; Visser et al., 2013; Kusum et al., 2014; Xian et al., 2015) and medical metabolites such as 3-*O*-methylfunicone and talaromycolides 1–3, 5, and 11. The metabolite 3-*O*-methylfunicone is able to repress mesothelioma cell motility, while talaromycolide kills human-pathogenic methicillin-resistant *Staphylococcus aureus* (Buommino et al., 2012; Zhai et al., 2015). *T. pinophilus* strain 1–95 was isolated in China (Xian et al., 2015), and shown to produce several PBDEs (Li et al., 2017), albeit at low yields.

Comparative transcriptomic profiling and genetic analyses of *T. pinophilus* strain 1–95 identified seven novel regulatory genes that regulate SSDE production. Among them, TpRfx1 (TP06128), positively regulated SSDE production of *T. pinophilus* via binding to the promoter regions of major amylase genes (Liao et al., 2018). Intriguingly, deletion of another candidate regulatory gene *TP05746*, encoding a Zn2Cys6 protein, resulted in a 51.4% increase in SSDE production, compared with the parental strain Δ*TpKu70* when cultured directly on medium containing soluble corn starch (SCS) for 5 days (Liao et al., 2018), but its detailed biological roles are unknown.

In this study, we found that *TP05746* regulated the production of various PBDEs including SSDE, RSDE, cellulase and xylanase of *T. pinophilus*, as well as mycelial growth and conidiation. Experiments further confirmed that TP05746 could bind to the promoter regions of major PBDE genes and their key regulatory genes, and to growth- and development-associated regulatory genes.

## Materials and Methods

### Fungal Strains and Culture Conditions

*Talaromyces pinophilus* wild-type strain 1–95 (#2645, China General Microbiological Culture Collection, Beijing, China; CGMCC) was isolated from a dry ploughed field in China (Xian et al., 2015). Deletion mutants Δ*TpKu70* and Δ*TP05746* were constructed by knocking out the *TpKu70* gene in strain 1–95 and the *TP05746* gene in Δ*TpKu70*, respectively (Zhang et al., 2017; Liao et al., 2018). The Δ*TpKu70* has no apparent difference in vegetative growth and enzyme production when compared with wild-type strain 1–95 (Zhang et al., 2017). All *T. pinophilus* strains were maintained on potato-dextrose agar (PDA) plates at 4°C or stored in 25% glycerol at -80°C. Cultivation of *Penicillium oxalicum* strains was consistent with the *T. pinophillus* strains. Mutant Δ*PoxKu70* (#3.15650, CGMCC) was derived from the wild-type HP7-1 via deleting gene *PoxKu70* (Zhao et al., 2016).

Asexual spores (conidia) were collected from fungal cells cultured on PDA plates at 28°C for 6 days, resuspended in aqueous 0.2% (v/v) Tween-80, and adjusted to a concentration of 1 × 10^8^ spores mL^–1^. For mycelial growth for DNA extraction, the fungus was cultured on liquid complete medium (LCM; Liao et al., 2018). Culture conditions for enzyme activity analysis, RNA sequencing, and real-time quantitative reverse transcription-PCR (RT-qPCR) analysis were as described previously (Liao et al., 2018). For RNA sequencing, total RNA was extracted from mycelium harvested after 12-h culture following transfer from glucose into medium containing 1% (w/v) soluble corn starch (SCS; Sigma-Aldrich, St. Louis, MO, United States).

For mycelial growth and observation by light microscopy, standard liquid medium (SLM; Liao et al., 2018) containing 1% (w/v) D-glucose, 1% (w/v) SCS, 2% (w/v) wheat bran plus Avicel [WA; wheat bran: Avicel = 1: 1; (w/w); Sigma-Aldrich, St.] or 2% (w/v) Avicel and/or 0.2% (w/v) 2-deoxy-glucose (2-DG) was inoculated with *T. pinophilus* spores and cultured at 28°C for 12 to 120 h. For phenotypic analyses, 1% (w/v) D-glucose, 1% (w/v) SCS or 2% (w/v) WA were added to solid low-salt minimal medium (LsMM) plates, with PDA being used as a positive control. For measurement of enzymatic activity, *P. oxalicum* strains were cultivated in Avicel medium according to the descried by Yan et al. (2017).

### Extraction of Total DNA and RNA From *Talaromyces pinophilus*

Extraction of total DNA and RNA from mycelia of *T. pinophilus* strains was carried out by the method described previously (Yan et al., 2017) with some modifications. In brief, for total DNA extraction, the harvested mycelia were ground in liquid nitrogen and lysate reagent (pH 8.0) was added immediately at a ratio of 10:1 (v: w). Subsequently, an equal volume of a phenol-chloroform mixture was added to the extract to remove proteins, followed by centrifuging at 11,300 × *g* at 4°C for 10 min. Finally, DNA was precipitated using isopropanol at a ratio of 1:1 (v: v).

Total RNA was extracted using TRIzol RNA Kit (Life Technologies, Carlsbad, CA, United States), according to the manufacturer’s instructions.

### Southern Hybridization Analysis

The only one-site mutation in the mutant Δ*TP05746* was confirmed using Southern hybridization analysis, according to the protocols of the DIG (digoxigenin)-High Prime DNA Labeling & Detection Starter Kit (Life Technologies, Carlsbad, CA, United States). Briefly, genomic DNA from each of the Δ*TpKu70* and the Δ*TP05746* mutants was extracted and digested by *Hin*dIII (TaKaRa Bio Inc., Dalian, China), and then transferred onto a Hybond-N^+^ Nylon membrane (GE Healthcare Limited, Amersham, United Kingdom). PCR was used to amplify the hybridization probe, using a specific primer pair TP05746-T-F/TP05746-T-R (Supplementary Table S1).

### Measurement of PBDE Production and Intracellular Protein Concentration Assays

Plant-biomass-degrading enzymes activities, including SSDE, raw starch-digesting enzymes (RSDE), cellulase and xylanase activities, were assayed as previously described (Liao et al., 2018; Zhang et al., 2019). Briefly, 50 μL of appropriately diluted crude extract produced from the parental strain Δ*TpKu70* or its deletion mutant Δ*TP05746*, were added to 450 μL of 100 mM citrate-phosphate buffer (pH 5.0) containing 1% SCS (Sigma-Aldrich), 1% raw cassava flour (farmer’s market, Nanning, China), 1% CMC-Na (Sigma-Aldrich, Darmstadt, Germany), or 1% beechwood xylan (Megazyme International Ireland, Bray, Ireland), and 1 mL of 100 mM citrate-phosphate buffer pH 5.0, containing Whatman No. 1 filter paper (50 mg, 1.0 cm × 6.0 cm; GE Healthcare Limited, Little Chalfont, United Kingdom). The mixture was incubated at 50–60°C for 10–60 min. The reducing sugars released were determined using the 3,5-dinitrosalicyclic acid method, measuring A_540_ (Miller, 1959). One unit of enzymatic activity (U) was defined as the required amount of enzyme required to produce 1 μmol reducing sugar per minute from the reaction substrates.

The substrates *p*-nitrophenyl-β-D-cellobioside (pNPC) and *p*-nitrophenyl-β-D-glucopyranoside (pNPG) (both from Sigma-Aldrich) were used for measurement of pNPCase and pNPGase activities, respectively. An aliquot (10 μL or 68 μL) of appropriately diluted crude extract from Δ*TpKu70* or Δ*TP05746*, was added into 130 or 72 μL of a mixture containing 14 μL 25 mM pNPG or pNPC, and incubated at 50°C for 15 min. Sodium carbonate (70 μL, 0.4 M) was added to stop the reaction. The *p*-nitrophenol liberated was measured at wavelength of 410 nm, with one unit of enzymatic activity (U) being defined as the amount of enzyme that produced 1 μmol of *p*-nitrophenol per minute from an appropriate substrate. All the assays were performed in three biological replicates.

Protein concentration in extracts of *T. pinophilus* strains was measured using the Bradford assay kit (Pierce Biotechnology, Rockford, IL, United States).

### Determination of Mycelial Dry Weight

Mycelial dry weight of *T. pinophilus* strains cultured in SLM containing 1% D-glucose or 1% SCS as the sole carbon source was determined gravimetrically as previously described (Liao et al., 2018). In brief, 1.0 × 10^8^ fresh spores were inoculated into 100 mL of the above medium and shake-cultured at 28°C and 180 rpm for 12 to 84 h. The hyphae were harvested by vacuum suction filtration every 12 h and then dried at 50°C to a constant weight. All the assays were performed in three biological replicates and the mean ± SD of the three replicates is presented.

### Light Microscopy Observation

Light microscopy observation of *T. pinophilus* mycelia was performed according to previously described methods (Xiong et al., 2018). The harvested hyphae from *T. pinophilus* strains grown on SLM containing 1% D-glucose, 1% SCS, or 2% WA for 12–36 h was transferred onto microscope slides. The slides were observed under a light microscope (OLYMPUS DP480; Olympus, Tokyo, Japan), and photomicrographs were analyzed using cellSence Dimension digital imaging software (Olympus).

### Real-Time Quantitative Reverse Transcription-PCR (RT-qPCR) Analysis

The PrimeScript^TM^ RT Reagent kit (TaKaRa Bio Inc.) was used to synthesize the first-strand cDNA from total RNA of mutant Δ*TpKu70* as the template, according to the manufacturer’s instruction. The PCR reaction mixture (20 μL) was composed of 10 μL of SYBR Premix ExTaq II (TaKaRa Bio Inc), 1.6 μL of 10 μM each primer (Supplementary Table S1), 2.0 μL of first-strand cDNA and 6.4 μL of sterile water. All the reaction cycles were run as follows: initial denaturation for 3 min at 96°C, followed by 40 cycles of 10 s each at 96°C, and 60°C for 30 s. The fluorescence signals were observed at the end of each extension step at 80°C. The relative expression levels of the tested genes were calculated as described previously (Zhang et al., 2017).

### RNA Sequencing

RNA sequencing of *T. pinophilus* strains was performed by BGI, Shenzhen, China, as described by He et al. (2018). A cDNA library was constructed with an average length of 100 bp for each sample and assessed using an Agilent 2100 Bioanalyzer (Agilent Technologies, Santa Clara, CA, United States) and an ABI StepOnePlus real-time PCR system (Applied Biosystems, Foster City, CA, United States), and then sequenced using an Illumina HiSeq 4000 system. Clean reads generated were mapped onto the genome of *T. pinophilus* strain 1–95 (Li et al., 2017) for functional annotation using BWA v0.7.10-r789 (Li and Durbin, 2009) and Bowtie2 v2.1.0 (Langmead and Salzberg, 2012). The gene expression levels (fragments per kilobase of exon per million mapped reads, FPKM) and differentially expressed genes (DEGs) were calculated and screened for, using the software RAEM v1.2.12 (Li and Dewey, 2011) and DESeq (Love et al., 2014), respectively. Genes with | log2 (Δ*TP05746*_FPKM/Δ*TpKu70*_FPKM)| ≥ 1 and *p*-value ≤ 0.05 were defined as DEGs.

### Overexpression of Gene *TP05746* in *Penicillium oxalicum*

Overexpression of gene *TP05746* was performed in filamentous fungus *P. oxalicum* according to the method for complementary strain construction described by Yan et al. (2017). Briefly, the overexpression cassette comprised of four fragments including approximately 2 kb of left- and right-flanking sequences of gene *POX05007* encoding a aspartic protease (Wang et al., 2018), G418 resistance gene, and gene *TP05746* sequence with a strong promoter pPoxEgCel5B (Wang et al., 2018) and gene *TP05586* terminator, was constructed via fusion PCR. These fragments were amplified using PCR with specific primer pairs (Supplementary Table S1). The constructed overexpression cassette was introduced into the fresh protoplasts of the parental strain Δ*PoxKu70* based on the methods previously described (Zhao et al., 2016). The overexpressed transformants were isolated and confirmed via corresponding antibiotics G418 and hygromycin, and PCR with specific confirmation primers (Supplementary Table S1).

### Recombinant Expression of DNA Fragment Encoding TP05746

A DNA fragment (1002 bp) of *TP05746* was amplified with PCR using primer pairs TP05746-F2/TP05746-R2 (Supplementary Table S1). The DNA fragment was cloned into vector pET32a (+) digested with *Sac*I, using ClonExpress^®^ II One Step Cloning Kit (Vazyme Biotech, Nanjing, China). The recombinant plasmid pET32a-TP05746 was introduced into *E. coli* Trans-DE3 cells, and then induced with 0.5 mM isopropyl-beta-D-thiogalactopyranoside (IPTG) and cultured at 16°C for 24 h. The recombinant protein, rTP05746, labeled with Trx-His-S tags, was purified using Ni-nitrilotriacetic acid (Ni-NTA) resin. Trx-His-S fusion protein and bovine serum albumin (BSA) were used as negative controls.

### Electrophoretic Mobility Shift Assay

Electrophoretic mobility shift assay (EMSA) was performed as previously described (Yan et al., 2017). Briefly, DNA fragments approximately 1,000-bp upstream from the ATG start codons of the tested genes were amplified by PCR, using specific primers labeled with 6-carboxyfluorescein (FAM) at the 3′ terminus (Supplementary Table S1) as EMSA probes, and a 500-bp DNA sequence upstream from the ATG start codon of *TP10751* encoding β-tubulin (with a FAM label) was used as a control. DNA fragments of the same length but without the FAM label were used as competitive EMSA probes. Approximately 50 ng of DNA probes were mixed with 0, 0.5, 1.0, 1.5, or 2.0 μg of rTP05746 in 2 μL of binding buffer (Liao et al., 2018) and incubated at 25°C for 20 min. In each EMSA reaction, non-specific sheared salmon sperm DNA was added, in order to prevent non-specific binding between protein and probes. For the control, 2.0 μg of BSA or Trx-His-S fusion protein were used. For the competitive EMSA, 2.0 μg of rTP05746 were mixed with 50 ng of DNA probe with the FAM label and 250–2500 ng of competitive EMSA probes. DNA-protein complexes were separated by 4% polyacrylamide Tris–acetic acid–EDTA gel electrophoresis and detected with the Bio-Rad ChemiDoc^TM^ MP Imaging System (Bio-Rad Laboratories, Inc., Hercules, CA, United States) at 489–506 nm.

### Phylogenetic Analysis

Proteins homologous to TP05746 were downloaded from NCBI website using BLASTP^2^. The neighbor-joining method and a Poisson correction model were used to construct a phylogenetic tree using software MEGA7.0 (Kumar et al., 2016), with 1000 replicates being used to calculate bootstrap values and gaps, and to handle missing data.

### Statistical Analysis

All experimental data associated with enzyme production, intracellular protein concentration, counts by microscopy, gene transcription and biomass were statistically analyzed using Microsoft Excel (Office 2016; Microsoft, Redmond, WA, United States). Summary statistics presented are mean ± SD. Significance analyses (*p* ≤ 0.05 or *p* ≤ 0.01) among samples were performed using Student’s *t* test.

### Accession Numbers

The DNA sequence of *TP05746* is available from the GenBank database under accession number MH447996. The transcriptomic data of *T. pinophilus* strains have been deposited in Gene Expression Omnibus (GEO) on NCBI (accession No. GSE131872).

## Results

### TP05746 Regulates the Production of PBDEs in *Talaromyces pinophilus*

**FIGURE 1:**
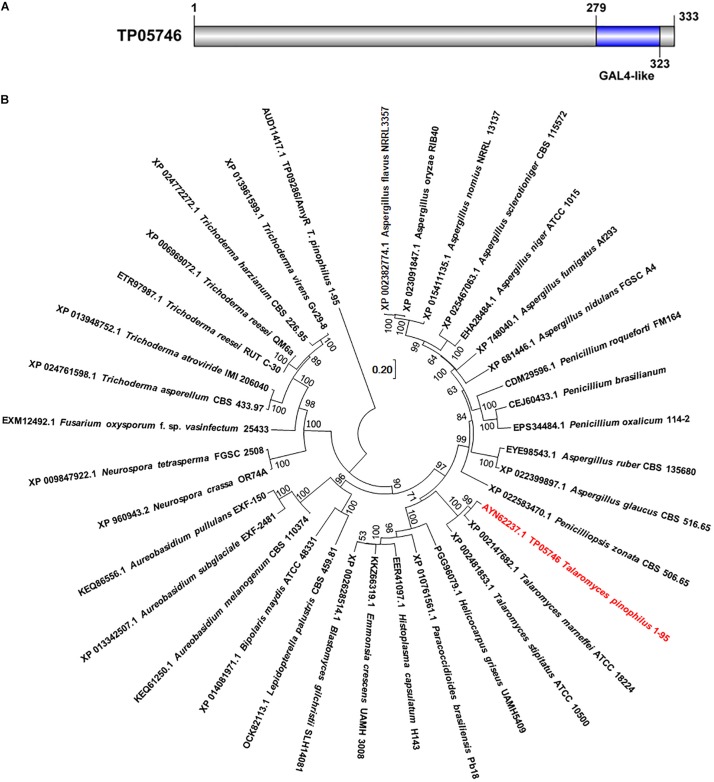
Characterization of TP05746 from *Talaromyces pinophilus*. **(A)** Conserved domain in TP05746. **(B)** Phylogenetic analyses of TP05746 and its homologous proteins. The cladogram was constructed by software MEGA7 based on the neighbor-joining method and a Poisson correction model. Scale bar indicates branch lengths.

Previous work had initially found that a deletion mutant Δ*TP05746* of *T. pinophilus* showed a 51.4% increase in SSDE production, compared with the parental strain Δ*TpKu70* cultured directly in medium containing SCS as the sole carbon source (Liao et al., 2018). Protein TP05746, composed of 333 amino acids, contains a conserved Gal4-like domain, also known as a Zn2Cys6 zinc finger domain, at its C-terminus, as identified through NCBI BlastP query (Figure 1A). TP05746 shared 87% and 43% of identities with putative C6 transcription factor PMAA_081800 in *Talaromyces marneffei* ATCC 18224 (XP_002147682.1) and AN8177.2 in *Aspergillus nidulans* FGSC A4 (XP 022399897.1), respectively, with the coverage of 100% and 83%, respectively, whereas most of the aligned proteins were selected based on the conserved Zn2Cys6 zinc finger domain at the C-terminus. Additionally, phylogenetic analyses demonstrated that the TP05746 and its homologs were specific to *Talaromyces*, and phylogenetically close to those in the Ajellomycetaceae (Figure 1B).

Southern hybridization analysis was employed to confirm the identity of the mutant Δ*TP05746*. The results showed that an expected 6.3-kb band appeared in the transformants of Δ*TP05746*, compared with a 3.2-kb band for Δ*TpKu70* (Supplementary Figure S1), confirming that a *TP05746* deletion cassette inserted into only the one correct site in the fungal genome. PCR analysis also confirmed that the deletion cassette replaced the *TP05746* locus (Liao et al., 2018).

**FIGURE 2:**
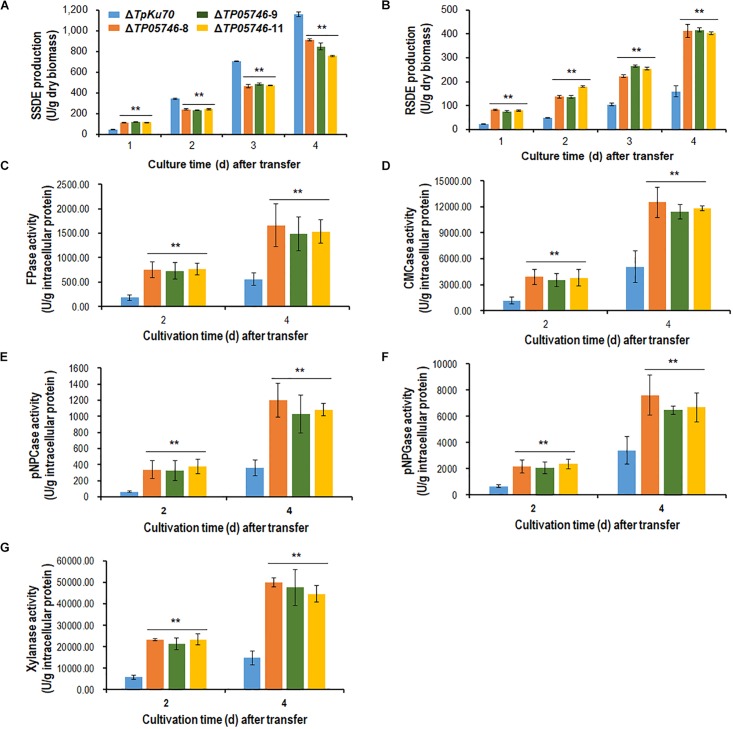
Plant-biomass-degrading enzymes production by *T. pinophilus* mutant Δ*TP05746* and the parental strain Δ*TpKu70*. Crude extracts were produced from *T*. *pinophilus* strains cultured after a transfer from glucose medium to SCS medium **(A,B)** or WA medium **(C–G)** for 1–4 days. **(A)** SSDE production. **(B)** RSDE production. **(C)** FPase production. **(D)** CMCase production. **(E)** pNPCase production. **(F)** pNPGase production. **(G)** Xylanase production. All experiments were performed with three independent biological replicates. Each data point is mean ± SD. ^∗∗^*p* ≤ 0.01 indicates differences between the deletion mutant Δ*TP05746* and the parental strain Δ*TpKu70* by Student’s *t-*test. PBDE, plant-biomass-degrading enzymes; SSDE, soluble-starch-degrading enzymes; SCS, soluble corn starch; RSDE, raw-starch-degrading enzymes; FPase, filter paper cellulase; CMCase, carboxymethylcellulase; pNPCase, *p*-nitrophenyl-β-cellobiosidase; pNPGase, *p*-nitrophenyl-β-glucopyranosidase.

*Talaromyces pinophilus* mutant strain Δ*TP05746* and its parental strain Δ*TpKu70* were then precultured in glucose medium for 24 h, and the same amounts of mycelia were transferred into fresh SLM containing SCS as carbon source and cultured for 1–4 days. Enzyme activity tests indicated that Δ*TP05746* exhibited 149.6% higher SSDE production at day one of SCS induction but then 27.6–33.1% decrease at days 2–4, relative to that exhibited by Δ*TpKu70* (*p* < 0.01, Student’s *t-*test; Figure 2A). Intriguingly, the RSDE production of Δ*TP05746* increased by 136.8–240.0% over the entire culture period (*p* < 0.01, Student’s *t-*test; Figure 2B).

In addition to amylase production, cellulase and xylanase production of the Δ*TP05746* were also tested on WA for 2–4 days, using the parental strain Δ*TpKu70* as a control. The results indicated that the production of cellulase, including FPase, CMCase, pNPCase, pNPGase, and xylanase in Δ*TP05746* increased to various degrees, ranging from 90.1 to 519.1%, depending on the enzyme class, compared with those in Δ*TpKu70* (*p* < 0.01, Student’s *t-*test; Figures 2C–G).

### *TP05746* Controls Conidiation of *Talaromyces pinophilus*

**FIGURE 3:**
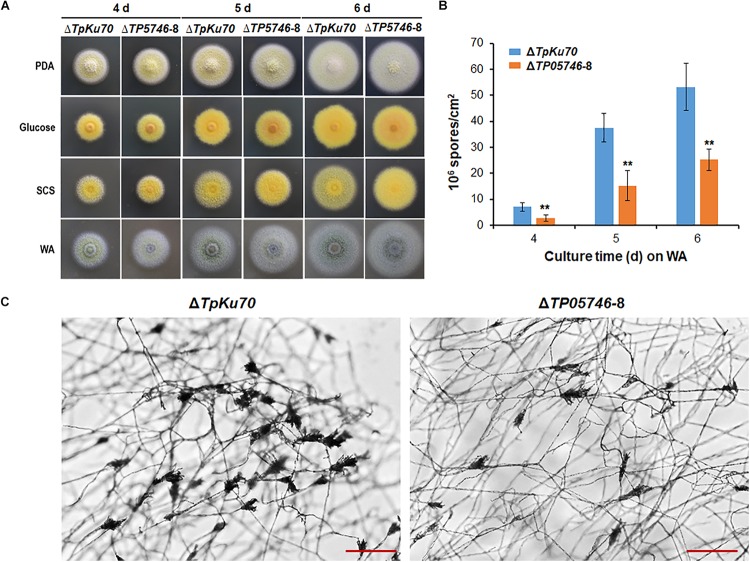
Phenotypic analyses of *T. pinophilus* mutant Δ*TP05746* and the parental strain Δ*TpKu70*. **(A)** Colonies on solid LsMM plates containing glucose, SCS, or WA, with PDA being used for a positive control. Fungal strains were incubated at 28°C for 4–6 days. **(B)** Numbers of asexual spores on WA. Each data point is mean ± SD. ^∗∗^*p* < 0.01 indicates differences between the Δ*TP05746* and parental strain Δ*TpKu70* by Student’s *t* test. **(C)** Microscopic investigation of conidiophores. Fungal strains were cultivated on WA at 28°C for 36 h. The mutant Δ*TP05746* exhibited fewer phialides in comparison with the parental strain Δ*TpKu70*. LsMM, low-salt minimal medium; SCS, soluble corn starch; WA, wheat bran plus Avicel; PDA, potato dextrose agar.

When both strains, Δ*TP05746* and Δ*TpKu70*, were inoculated onto solid LsMM containing D-glucose, SCS or WA, with PDA being used as a control, colony phenotypes were observed. As shown in Figure 3A, no significant difference was found in the colony size between Δ*TP05746* and Δ*TpKu70* on any of the above-mentioned plates, while colony color of Δ*TP05746* was slightly different from that of Δ*TpKu70* on all plates.

Asexual spores (conidia) produced by Δ*TP05746* were then counted following inoculation onto WA plates, using the hemocytometer method (Christensen et al., 2012). The results revealed that Δ*TP05746* produced 52.6–61.9% of (*p* < 0.01, Student’s *t-*test) spores relative to Δ*TpKu70* at 4–6 days (Figure 3B).

As shown in Figure 3C, microscopic investigation showed that the mutant Δ*TP05746* exhibited delayed phialide development compared with the parental strain Δ*TpKu70* on WA-supplemented solid LsMM plates.

### *TP05746* Accelerates Growth of *Talaromyces pinophilus* at Early Stage of SCS or WA Induction but Delays Growth at Later Stages

**FIGURE 4:**
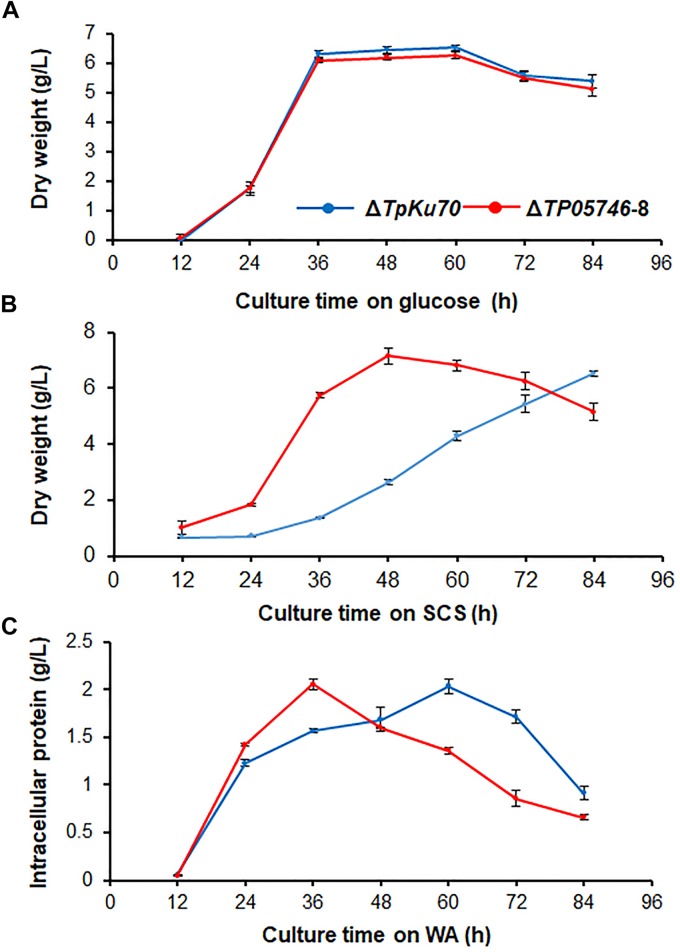
Growth curves of *T. pinophilus* mutant Δ*TP05746* and the parental strain Δ*TpKu70* in SLM containing glucose **(A)**, SCS **(B)**, or WA **(C)** as the sole carbon source for 12–84 h at 28°C, respectively. Three independent experiments were performed as biological replicates. SLM, standard liquid medium; SCS, soluble corn starch; WA, wheat bran plus Avicel.

To investigate whether *TP05746* affected the growth of *Talaromyces pinophilus*, real-time quantitative growth curves in the presence of glucose, SCS or WA of both Δ*TP05746* and Δ*TpKu70* were determined and then compared. The results revealed that Δ*TP05746* produced mycelial dry weights similar to Δ*TpKu70* in the glucose medium (Figure 4A). When using SCS or WA instead of glucose, however, Δ*TP05746* grew faster (*p* < 0.01, Student’s *t-*test) than Δ*TpKu70* at the early induction period (i.e., 24–36 h for WA, and between 24–60 h for SCS) but the growth rate of Δ*TP05746*, compared with Δ*TpKu70*, fell sharply at the later stages (*p* < 0.05, Student’s *t-*test; Figures 4B,C).

**FIGURE 5:**
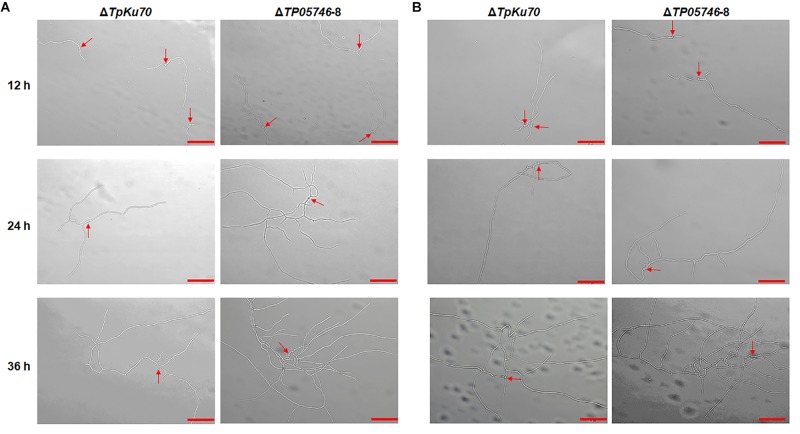
Microscopic investigation of growth of *T. pinophilus* mutant Δ*TP05746* and the parental strain Δ*TpKu70* on SLM containing SCS **(A)** or WA **(B)**. Fungal strains were cultured at 28°C for 12–36 h. The mutant Δ*TP05746* cultured on SCS or WA for 24–36 h showed more hyphal branches than that of the parental strain Δ*TpKu70*. Red arrows indicate the developing conidia. Bar is 50 μm. SLM, standard liquid medium; SCS, soluble corn starch; WA, wheat bran plus Avicel.

Further microscopic observations revealed that the mutant Δ*TP05746* exhibited significantly more hyphal branching when compared with the parental strain Δ*TpKu70* at 24 and 36 h in both liquid SCS and WA medium, although there was no significant difference at 12 h (Figure 5). Moreover, mycelial development of the Δ*TP05746* was the same as that of the Δ*TpKu70* when cultivated on liquid glucose medium (Supplementary Figure S2).

### Transcriptomic Analyses Reveal That *TP05746* Regulates the Expression of Genes Encoding PBDEs and Their Regulators, and Hyphal Development-Associated Genes in *Talaromyces pinophilus*

**FIGURE 6:**
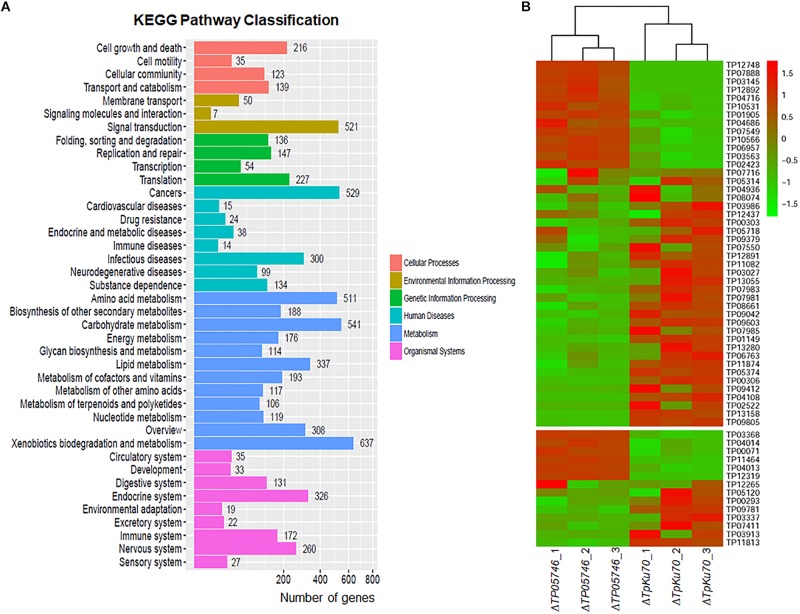
Transcriptomic analysis of *T. pinophilus* mutant Δ*TP05746* and the parental strain Δ*TpKu70* cultured in SLM containing SCS as the sole carbon source for 12 h. **(A)** KEGG pathway classification annotation of 4429 DEGs in the Δ*TP05746* [a | log2(fold change)| ≥ 1 and a *p*-value ≤ 0.05 as thresholds]. **(B)** Plant cell-wall-degrading enzymes and amylase genes regulated by *TP05746*. SLM, standard liquid medium; SCS, soluble corn starch; KEGG, kyoto encyclopedia of genes and genomes; DEGs: differently expressed genes.

The genome-wide mRNA abundance of both the parental strain Δ*TpKu70* and its mutant Δ*TP05746* were measured in cultures grown in SLM containing SCS as the sole carbon source for 12 h. Comparative transcriptomics identified 4429 differentially expressed genes (DEGs) in mutant Δ*TP05746*, using a | log2 (fold change)| ≥ 1 and *p*-value ≤ 0.05 as thresholds relative to expression in the parental strain Δ*TpKu70* (Supplementary Table S2). Among them, the transcripts of 2470 genes were up-regulated and 1959 were down-regulated. Kyoto Encyclopedia of Genes and Genomes (KEGG) annotation revealed that the DEGs were mainly involved in metabolism (47%), with the others being involved in human diseases (15%), organismal systems (15%), environmental information processing (8%), genetic information processing (8%), and cellular processes (7%) (Figure 6A). Moreover, the number of up-regulated genes involved in metabolism was greater than that of down-regulated genes (Supplementary Figure S3).

Of the DEGs, 14 were involved in starch degradation, including three *amy* genes *TP03368*, *TP04014*/*Amy13A* and *TP07411*, one *gla* gene *TP12319*, and ten *aga* genes *TP00071*, *TP00293*, *TP03337*, *TP03913*, *TP04013*, *TP05120*, *TP09781*, *TP11813*, *TP11464*, and *TP12265*. Comparative analyses revealed that the transcripts of six of these 14 starch-degradation-associated DEGs (*TP03368*, *TP04014*/*Amy13A*, *TP12319*, *TP00071*, *TP04013*, and *TP11464*) were upregulated in Δ*TP05746* in comparison with the expression level in Δ*TpKu70*, with a log2 fold change ranging from 1.27 to 5.90. The other eight DEGs were downregulated, with a log2 fold change ranging from −5.46 to −1.42 (Figure 6B).

Interestingly, 44 of the DEGs included in the *TP05746* regulon encoded plant cell wall-degrading enzymes (CWDEs), including one *cbh* gene (*TP09412*/*cbh1*), two *eg* genes (*TP04686* and *TP06957*), and 13 *bgl* genes (*TP01149*, *TP02423*, *TP04716*, *TP05374*, *TP07549*, *TP07716*, *TP07981*, *TP07983*, *TP08074*, *TP09042*, *TP09603*, *TP11082*, and *TP12437*). Of these 44 CWDE-encoding DEGs, 13 genes (*TP01905*, *TP02423*, *TP03145*, *TP03563*, *TP04686*, *TP04716*, *TP06957*, *TP07549*, *TP07888*, *TP10531*, *TP10566*, *TP12748*, and *TP12892*) were upregulated in Δ*TP05746* compared with Δ*TpKu70*, with a log2 fold change ranging from 1.51 to 5.93, whereas the other 31 genes were down-regulated, with a log2 fold change ranging from −7.83 to −1.13 (Figure 6B).

In addition to genes encoding carbohydrate-degrading enzymes, the 377 DEGs encoding putative TFs were found, most of which contained Zn2Cys6, C2H2, bZIP, winged helix repressor or homeodomain-like domains, consisting of 159 upregulated (1.03 < log2 fold change < 9.81) and 218 down-regulated (−10.68 < log2 fold change < −1.00) DEGs (Supplementary Table S2). Notably, six regulatory genes, known to be involved in controlling the expression of genes encoding PBDEs, were detected, including *TP08849/AreA* (log2 fold change = 3.45), *TP09286/AmyR* (log2 fold change = 1.10), *TP10486/ClrB* (log2 fold change = 5.52), *TP02627/XlnR* (log2 fold change = −1.53), *TP00292/BglR* (log2 fold change = 3.89), and *TP06351/Vib1* (log2 fold change = −3.22) (Li et al., 2017).

Previous work had shown that deletion of *TP05746* resulted in a reduction in asexual spore production, suggesting that *TP05746* controls conidiation of *T. pinophilus*. In the DEGs, 13 genes (*TP04427/Bud4*, *TP10226/Asp*, *TP10901/VosA*, *TP03893/FadA*, *TP04237/NimX*, *TP05733/WetA*, *TP11170/FlbA-*like gene, *TP10098/FlbA*, *TP03987/VeA*, *TP05897/RodA*, *TP05092/ArpA, TP13250/ArpA*-like gene, and *TP06809/ArpA*-like gene) were observed, which were previously reported to be involved in fungal conidiogenesis (Qin et al., 2013), of which five (*TP04427/Bud4*, *TP10226/Asp*, *TP10901/VosA*, *TP03893/FadA*, and *TP04237/NimX*) were upregulated (1.67 < log2 fold change < 4.73) and eight were downregulated (−6.55 < log2 fold change < −1.19) in Δ*TP05746* (Supplementary Table S2).

### RT-qPCR Shows That *TP05746* Dynamically Controls the Expression of Genes Encoding Major PBDE Genes and Their Regulators, and Hyphal Development-Associated Genes in *Talaromyces pinophilus*

**FIGURE 7:**
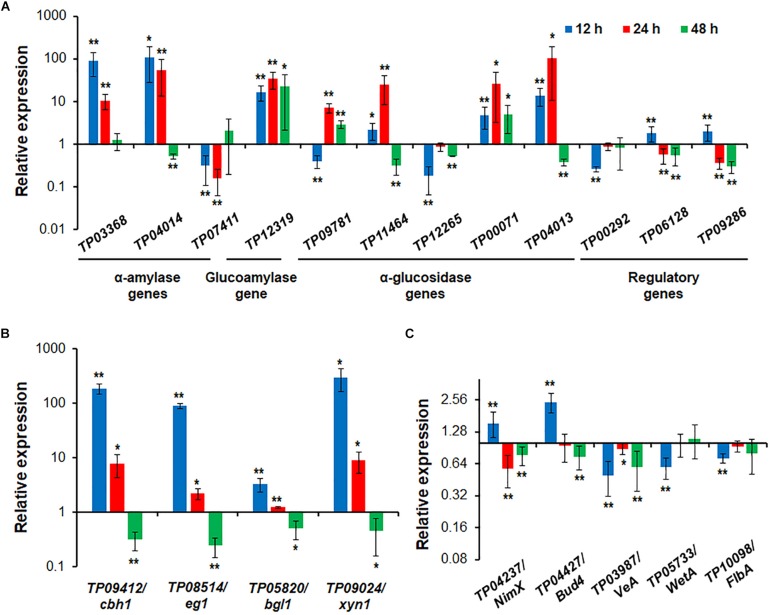
Expressional regulation of enzyme genes and regulatory genes by *TP05746* in *T. pinophilus* as demonstrated by RT-qPCR. **(A)** Amylase genes and their regulatory genes. Total RNA was extracted from fungal strains cultured in medium containing SCS as the carbon source for 12, 24, and 48 h after transfer from glucose. **(B)** Cellulase and xylanase genes. **(C)** Genes involved in conidiogenesis. Fungal cells were cultured on WA for 12, 24, and 48 h after transfer from glucose. Expression levels of the genes tested in the Δ*TP05746* mutant were normalized against the parental strain Δ*TpKu70*. ^∗∗^*p* ≤ 0.01 and ^∗^*p* ≤ 0.05 indicate differences between Δ*TP05746* and Δ*TpKu70* by Student’s *t* test. Each experiment contained three biological replicates. RT-qPCR, real-time quantitative reverse transcription PCR; WA, wheat bran plus Avicel.

RT-qPCR was employed to investigate regulatory dynamics by *TP05746* of the expression of genes involved in the degradation of plant biomass in *T. pinophilus*. On the basis of comparative transcriptomic data, the target genes were identified, including three *amy* genes *TP03368*, *TP04014*/*Amy13A* and *TP07411*, one *gla* gene *TP12319*, and five *aga* genes *TP09781*, *TP11464*, *TP12265*, *TP00071* and *TP04013*, as well as their known regulatory genes *TP09286/AmyR* (Zhang et al., 2017), *TP06128/Rfx1* (Liao et al., 2018), and *TP00292/BglR* (Xiong et al., 2017), and their expression (relative to the levels in the parental strain Δ*TpKu70*) was measured at 12, 24, and 48 h after SCS induction in the mutant Δ*TP05746*. The results showed that the transcript abundance of these 12 genes was significantly altered in Δ*TP05746* relative to the parental strain. At 12 h after induction, the expression of genes *TP03368*, *TP04014*/*Amy13A*, *TP12319*, *TP11464*, *TP00071*, *TP04013*, *TP09286/AmyR*, and *TP06128/Rfx1* in the mutant Δ*TP05746* exhibited significant up-regulation, ranging from 1.17- to 87.77-fold, whereas the expression of four genes, *TP07411*, *TP09781*, *TP12265*, and *TP00292/BglR*, was down-regulated by 59.7–81.9% in the mutant Δ*TP05746* (*p* < 0.05, Student’s *t-*test). At 24 h, all these targeted genes, except for *TP07411*, *TP12265*, *TP09286/AmyR*, *TP06128/Rfx1*, and *TP00292/BglR*, were significantly upregulated in Δ*TP05746*, the increase ranging from 614.8 to 10108.2% (*p* < 0.05, Student’s *t* test). The transcript abundance of *TP09286/AmyR*, *TP06128/Rfx1*, and *TP07411* decreased by 43.7 to 84.0%. By contrast, the transcript abundance of only three genes, namely *TP12319*, *TP09781*, and *TP00071*, continued to increase, by 190.0 to 2142.8% in Δ*TP05746* at 48 h. Six genes, *TP04014*, *TP11464*, *TP12265*, *TP04013*, *TP09286/AmyR*, and *TP06128/Rfx1* showed a 44.2–70.2% decrease in transcript abundance (*p* < 0.05, Student’s *t* test; Figure 7A).

In addition to starch-degrading enzyme genes, four cellulase and xylanase genes, *TP09412*/*cbh1*, *TP08514*/*eg1*, *TP05820*/*bgl1* and *TP09024*/*xyn1*, were also selected and their real-time transcription investigated in both Δ*TP05746* and Δ*TpKu70* cultured on WA. The results indicated that the expression of all the cellulase and xylanase genes tested was upregulated to various degrees in Δ*TP05746* relative to Δ*TpKu70* in the early induction period (before 24 h) but downregulated in the later induction period. For example, the transcription levels of all four genes increased by between 228.6% and 29565.0% at 12 h but by between 22.4% and 802.8% at 24 h (*p* < 0.05, Student’s *t-*test) in Δ*TP05746* (Figure 7B). Conversely, their expression was consistently downregulated by 50.4–75.6% in the mutant at 48 h (*p* < 0.05, Student’s *t-*test) (Figure 7B).

Five key genes involved in fungal conidiogenesis, namely *TP04427/Bud4*, *TP04237/NimX*, *TP05733/WetA*, *TP10098/FlbA*, and *TP03987/VeA*, were selected for RT-qPCR. The results indicated that, at 12 h, the transcripts of both *TP04237/NimX*, and *TP04427/Bud4* were significantly upregulated, by 54.8% and 143.7%, respectively, while expression of the others was downregulated by 27.5 to 50.0% (*p* < 0.01, Student’s *t-*test). At 24 h, the expression of two genes, *TP04237/NimX*, and *TP03987*/*VeA*, increased by 42.7% and 11.4%, whereas that of *TP04237/NimX*, *TP03987*/*VeA*, and *TP04427/Bud4* decreased by 23.1 to 40.7% (*p* < 0.05, Student’s *t-*test; Figure 7C).

### TP05746 Binds to the Promoter Regions of Genes Encoding Major PBDEs and Their Regulators *in vitro*, as Well as to Conidiogenesis-Involved Genes

**FIGURE 8:**
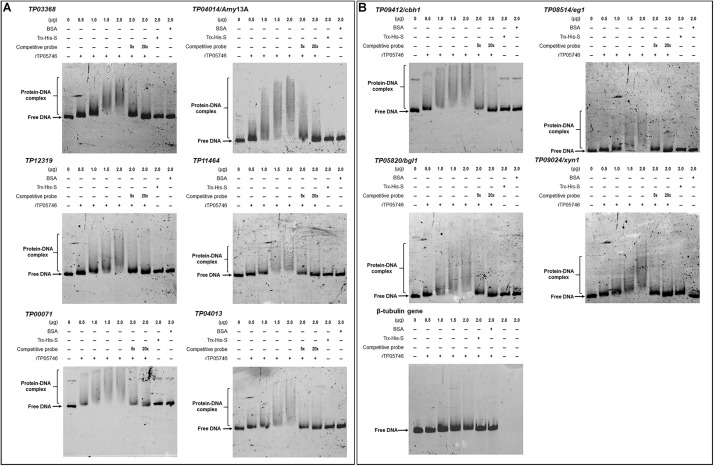
Interaction between TP05746 and genes encoding enzymes as revealed by electrophoretic mobility shift assay (EMSA). **(A)** Amylase genes. **(B)** Cellulase and xylanase genes. The recombinant protein rTP05746 (0–2.0 μg) was mixed with approximately 50 ng of FAM-labeled EMSA probes. EMSA probes lacking the FAM label were used for competitive EMSA. BSA, Trx-His-S fusion protein or the promoter region of the β-tubulin gene alone were used as controls. FAM, 6-carboxyfluorescein; EMSA, electrophoretic mobility shift assay; BSA, bovine serum albumin. In each EMSA reaction, non-specific sheared salmon sperm DNA was added, in order to prevent non-specific binding between protein and probes.

**FIGURE 9:**
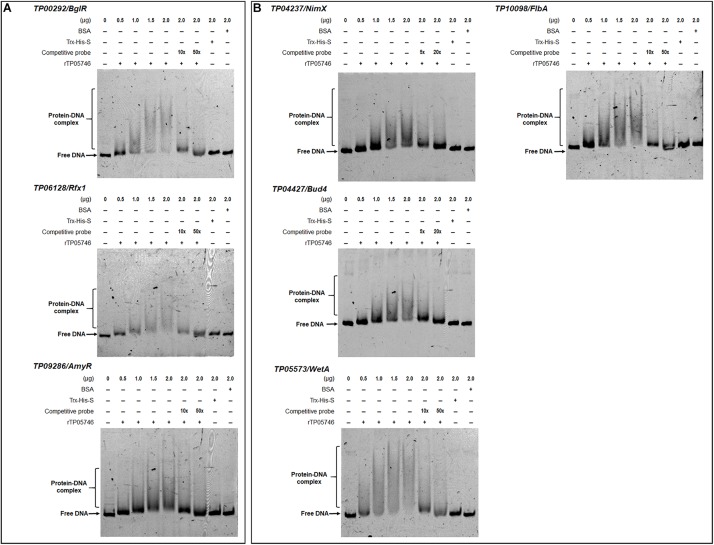
Interaction between TP05746 and regulatory genes revealed by electrophoretic mobility shift assay (EMSA). **(A)** Genes involved in the regulation of PBDE gene expression. **(B)** Genes involved in conidiogenesis. The recombinant protein rTP05746 (0–2.0 μg) was mixed with approximately 50 ng of FAM-labeled EMSA probes. EMSA probes lacking the FAM label were used for competitive EMSA. BSA, Trx-His-S fusion protein or the promoter region of the β-tubulin gene alone were used as controls. FAM, 6-carboxyfluorescein; EMSA, electrophoretic mobility shift assay; BSA: bovine serum albumin; PBDE, plant-biomass-degrading enzymes. In each EMSA reaction, non-specific sheared salmon sperm DNA was added, in order to prevent non-specific binding between protein and probes.

Electrophoretic mobility shift assay was used to confirm whether TP05746 directly or indirectly regulated the expression of target genes. The cDNA of *TP05746* was fused to a DNA fragment encoding Trx-His-S-tags and recombinantly expressed in *Escherichia coli*. The 6-carboxyflurescein (FAM)-tagged DNA probes (∼1,000 bp) in the promoter regions of two α-amylase genes *TP03368*, *TP04014*/*Amy13A*, one glucoamylase gene *TP12319*, three α-glucosidase genes *TP11464*, *TP00071*, *TP04013*, three cellulase genes *TP09412/cbh1*, *TP08514/eg1*, *TP05820/bgl1*, one xylanase gene *TP09024/xyn1*, and three regulatory genes *TP00292/BglR*, *TP06128/Rfx1*, and *TP09286/AmyR*, as well as four conidiogenesis-involved genes *TP04427/Bud4*, *TP04237/NimX*, *TP05733/WetA*, and *TP10098/FlbA*, were amplified using specific primer pairs (Supplementary Table S1). The promoter region of the β-tubulin gene *TP10751*, and either bovine serum albumin (BSA) or the Trx-His-S fusion protein was used as controls. DNA fragments of the same length but without the FAM label, were used as competitive probes. As shown in Figures 8, 9, shifted bands representing rTP05746-DNA complexes in all mixtures of the recombinant protein rTP05746 and EMSA probes of the target genes were observed to various degrees. Moreover, the band size gradually increased along with an increase in rTP05746 amounts (0–2.0 μg), whereas shifted bands did not occur between EMSA probes and either BSA or the Trx-His-S fusion protein, or between rTP05746 and the promoter region of the β-tubulin gene *TP10751*.

Simultaneously, competitive EMSA was performed using the competitive probes, and the results revealed that the concentration of the shifted bands gradually decreased in response to increasing amounts of the competitive probes without the FAM label (Figures 8, 9). However, in fact how interact with these promoters by *TP05746 in vivo* still awaits to be performed.

### TP05746 Inhibits the Production of PBDEs in Filamentous Fungus *Penicillium oxalicum*

**FIGURE 10:**
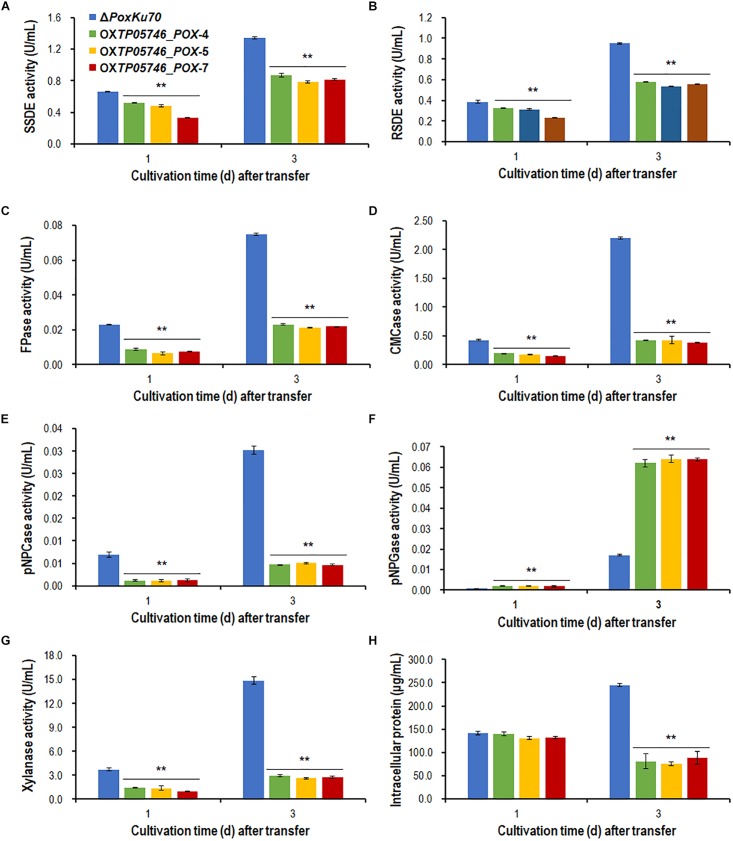
Plant-biomass-degrading enzymes production by overexpression strain OX*TP05746_POX* and the parental strain Δ*PoxKu70*. Crude extracts were produced from *Penicillium oxalicum* strains cultured after a transfer from glucose medium to Avicel medium for 1–3 days. **(A)** SSDE production. **(B)** RSDE production. **(C)** FPase production. **(D)** CMCase production. **(E)** pNPCase production. **(F)** pNPGase production. **(G)** Xylanase production. **(H)** intracellular protein. All experiments were performed independently for three biological replicates. Each data point is mean ± SD. ^∗∗^*p* ≤ 0.01 indicates differences between the deletion mutant Δ*TP05746* and the parental strain Δ*TpKu70* by Student’s *t* test. PBDE, plant-biomass-degrading enzyme; SSDE, soluble-starch-degrading enzyme; SCS, soluble corn starch; RSDE, raw-starch-degrading enzyme; FPase, filter paper cellulase; CMCase, carboxymethylcellulase; pNPCase, *p*-nitrophenyl-β-cellobiosidase; pNPGase, *p*-nitrophenyl-β-glucopyranosidase.

To further confirm the regulatory roles and potential application of gene *TP05746* in genetic engineering, gene *TP05746* was over-expressed in filamentous fungus *P. oxalicum* parental strain Δ*PoxKu70* (Supplementary Figure S4) derived from the wild-type HP7-1 via deleting gene *PoxKu70* (Zhao et al., 2016), and then its production of PBDEs was measured when cultivated on Avicel for 1–3 days. The results indicated the obtained overexpressed strain OX*TP05746*_*POX* lost 54.4–84.6% of cellulase (FPase, pNPCase and CMCase) and xylanase production, 15.6–49.9% of SSDE and RSDE production, while increased 131.1–275.1% of pNPGase production, compared with the parental strain Δ*PoxKu70* (*p* < 0.01, Student’s *t* test; Figures 10A–G). Intracellular proteins of strain OX*TP05746*_*POX* had no significant difference from that of the Δ*PoxKu70* when cultivated on WA for 1 day, but decreased by approximately 70% when for 3 days (*p* < 0.01, Student’s *t* test; Figure 10H).

## Discussion

**FIGURE 11:**
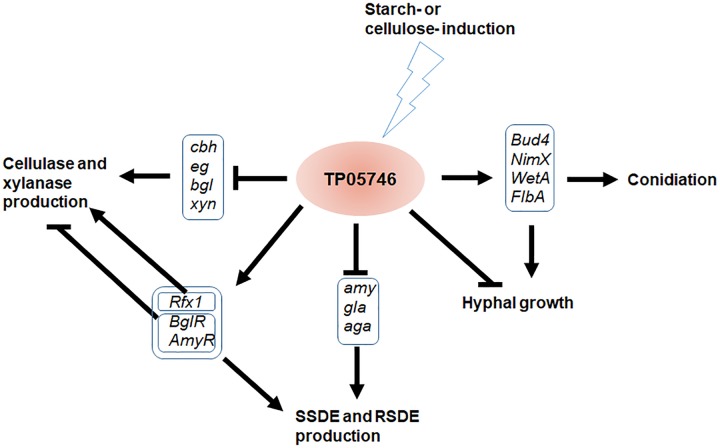
Proposed model of *TP05746* regulation in *T. pinophilus*. *cbh*, cellobiohydrolase gene; *eg*, endo-β-1,4-glucanase gene; *bgl*, β-1,4-glucosidase gene; *gla*, glucoamylase gene; *amy*, α-amylase gene; *aga*, α-glucosidase gene.

In this study, we explored regulatory roles of a novel Zn2Cys6 protein, TP05746, that regulated PBDE (i.e., SSDE, RSDE, cellulase, and xylanase) production as well as growth and positively regulated conidiation of *T. pinophilus* through regulating the expression of the associated genes and their regulatory genes (Figure 11). TP05746 specifically belonged to *Talaromyces*, and plays an essential regulatory role via a molecular mechanism distinct from that of the known CreA-mediated CCR.

The regulation of *TP05746* on PBDE production is time-dependent, as was expression of their encoded genes. For example, deletion of *TP05746* resulted in an increase in RSDE, cellulase and xylanase activity over the entire induction period, while the level of increasing production tended to fall over time, corresponding to dynamic changes in expression of major PBDE genes. The transcripts of *TP03368-*, and *TP04014/Amy13A-*encoding proteins that included SBDs in the mutant Δ*TP05746* first increased and then decreased, while the expression of *TP00071* continued to increase over time. Cellulase and xylanase genes *TP09412/cbh1*, *TP08514/eg1*, *TP05820/bgl1*, and *TP09024/xyn1* were repressed at the early induction stage but activated at later stages.

These observed phenomena are closely associated with substrate induction and a complex regulatory network. Plant biomass is degraded into single sugars via the synergistic action of several PBDEs working at appropriate respective rates, with the most-efficient enzyme mixture being secreted by the fungal cells based on which enzymes are needed in response to specific substrates. To the best of our knowledge, specific inducing substrates are low molecular weight sugars, in-process products of biomass degradation, such as cellobiose, xylobiose and maltose (Amore et al., 2013), but with unstable contents. Additionally, a complex regulatory network, consisting of various TFs and the target genes, are prerequisites to respond to the in-process products. As a note of this complex regulatory network, TP05746 regulates not only the expression of genes encoding specific enzymes, but also that of other TF genes, such as *AmyR*, *TpRfx1*, and *BglR*. AmyR and BglR positively regulate fungal amylase production and negatively control cellulase and xylanase production (Nitta et al., 2012; Li et al., 2015; Xiong et al., 2017; Zhang et al., 2017), whereas TpRfx1 positively mediates PBDE production, including amylase, cellulase and xylanase, in *T. pinopholus* (Liao et al., 2018). However, the actual regulatory mechanism of TP05746 in fungal cells still needs to be further elucidated.

In addition to fungal PBDE production, TP05746 also controls conidiation. Fungal conidiation is governed by the BrlA → AbaA → WetA regulatory cascade in concert with other genes, such as genes encoding FLBs (fluffy low *BrlA* expression) and the velvet family of proteins (Park and Yu, 2016). Here, this study found that deletion of *TP05746* affected the expression of several key conidiation-associated regulatory genes, such as *TP04427/Bud4*, *TP04237/NimX*, *TP05733/WetA*, *TP10098/FlbA* and *TP03987/VeA*. The gene *Bud4* is involved in spectrum formation in hyphal growth and the development of conidiophores in *Aspergillus*, deletion of which resulted in non-production of conidia (Si et al., 2012), as well as *FlbA* (Wieser et al., 1994). *NimX*, also called *cdc2*, encoding the cyclin-dependent kinase, accelerates hyphal branching and produces abnormal conidiophores at restrictive temperatures in *A. nidulans* (Lin and Momany, 2004). *WetA* is required for the middle to late phases of conidiation, to complete conidiation, and functions in the germination of these asexual spores and the early phase of mycelial growth (Tao and Yu, 2011). The velvet gene *VeA* is essential for proper asexual spore development but exhibits the opposite pattern of conidiation in *Aspergillus fumigatus* according to previous work (Dhingra et al., 2012; Park et al., 2012). Unfortunately, at this point it is not known how *TP03987/VeA* function in *T. pinophilus*. The expression of the above genes regulated by *TP05746* is time-dependent, and reaches a balance, resulting in the phenotypes observed in the mutant Δ*TP05746*. It should be noted that these conidiation-related genes might contribute to the regulation of cellulase and xylanase genes by *TP05746*. For example, the *VeA* ortholog *Vel1* of *T. reesei* positively regulates the expression of key cellulase and xylanase genes (Karimi Aghcheh et al., 2014).

In addition, *TP05746* represses hyphal growth of *T. pinophilus*, which might affect the utilization of nutrients. For example, under SCS induction, the expression of TP05746 is activated, thereby inhibiting the transcription of major amylase genes via a complex regulatory network, resulting in low yields of extracellular amylases. Starch could not be digested into small sugars, including glucose and maltose, to provide energy for the fungal cells.

To the best of our knowledge, few negative TFs have been identified in filamentous fungi to date, deletion of which results in an increase in PBDE production. The best-known repressor is CreA/Cre1/Cre-1, which inhibits almost all fungal genes involved in the degradation of plant biomass in the presence of the preferred carbon source, glucose, including both structural enzyme genes and regulatory genes (Portnoy et al., 2011; Sun and Glass, 2011; Li et al., 2015). Further experimental data revealed that, in the presence of D-glucose, both SSDE and RSDE production by Δ*TP05746* increased approximately 1.3- to 2.2-fold relative to that of the parental Δ*TpKu70.* In addition, 2-DG could activate CCR in the mutant Δ*TP05746*, leading to insufficient cellulase production, as in Δ*TpKu70* (Supplementary Figure S5). These data suggested that TP05746 was not involved in CreA-mediated CCR, suggesting that the mode of action of TP05746 is different from that of CreA.

We tried to construct the complementary strain of the mutant Δ*TP05746* at least four times but failed for unknown reasons. Three randomly chosen knock-out transformants for the gene *TP05746* in *T. pinophilus* were confirmed by both PCR with specific primers and by Southern hybridization analysis. Furthermore, in the enzymatic activity assay, all three randomly chosen transformants showed similar and consistent results, confirming that the obtained phenotype of each transformant was specifically generated by the deletion of the gene *TP05746*. Fortunately, gene *TP05746* was heterologously overexpressed in filamentous fungus *P. oxalicum*. Overexpression of *TP05746* resulted in the reduction of plant-biomass-degrading enzyme production, thereby leading to less accumulation of fungal hyphae. These data also confirmed that *TP05746* plays a negative role in the production of PBDEs.

It should be noted that negatively regulatory genes are potential targets for genetic engineering to achieve increased enzyme production. Deletion of *TP05746* led to a several-fold increase in RSDE production. RSDE application to starch biorefineries to generate biofuels or other biochemical products efficiently saves costs compared with traditional starch processing (Robertson et al., 2006; Görgens et al., 2015).

## Conclusion

In conclusion, the present study explored the regulatory roles of the novel TF TP05746 in *T. pinophilus* with respect to the control of PBDE production (SSDE, RSDE, cellulase and xylanase), as well as growth and conidiation. Further studies indicated that TP05746 dynamically regulated the expression of the associated genes described above, thereby affecting the corresponding fungal phenotypes. These findings provided novel insights into the regulatory mechanism of fungal PBDE gene expression and identify a potential target for genetic engineering for industrial application.

## Data Availability Statement

The datasets generated for this study can be found in the DNA sequence of TP05746 is available from the GenBank database under the accession number MH447996. The transcriptomic data of *T. pinophilus* strains have been deposited in Gene Expression Omnibus (GEO) on NCBI (accession no. GSE131872).

## Author Contributions

J-XF designed and supervised the study, and involved in data analysis and manuscript revision. SZ co-supervised all the experiments and revised the manuscript. TZ carried out the enzyme activity assay, phenotypic analysis, Southern hybridization analysis, RT-qPCR, and EMSA, and drafted the manuscript. L-SL involved in genomic DNA, RNA, and protein extraction and transcriptomic analysis. C-XL carried out the bioinformatic analysis. G-YL, XL, and X-ML took part in the preparation of experimental materials and data analysis. All authors read and approved the final manuscript.

## Conflict of Interest

The authors declare that the research was conducted in the absence of any commercial or financial relationships that could be construed as a potential conflict of interest.

## Footnotes

1 http://www.cazy.org/

2 https://blast.ncbi.nlm.nih.gov/Blast.cgi
