# Supplementary material for: Identification of a Novel Transcription Factor TP05746 Involved in Regulating the Production of Plant-Biomass-Degrading Enzymes in Talaromyces pinophilus
Source: Front Microbiol. 2019 Dec 13;10:2875. doi: 10.3389/fmicb.2019.02875 (PMC6923684; doi:10.3389/fmicb.2019.02875)
Supplement: Supplementary file 1 [file Data_Sheet_1.pdf]

Supplementary Material

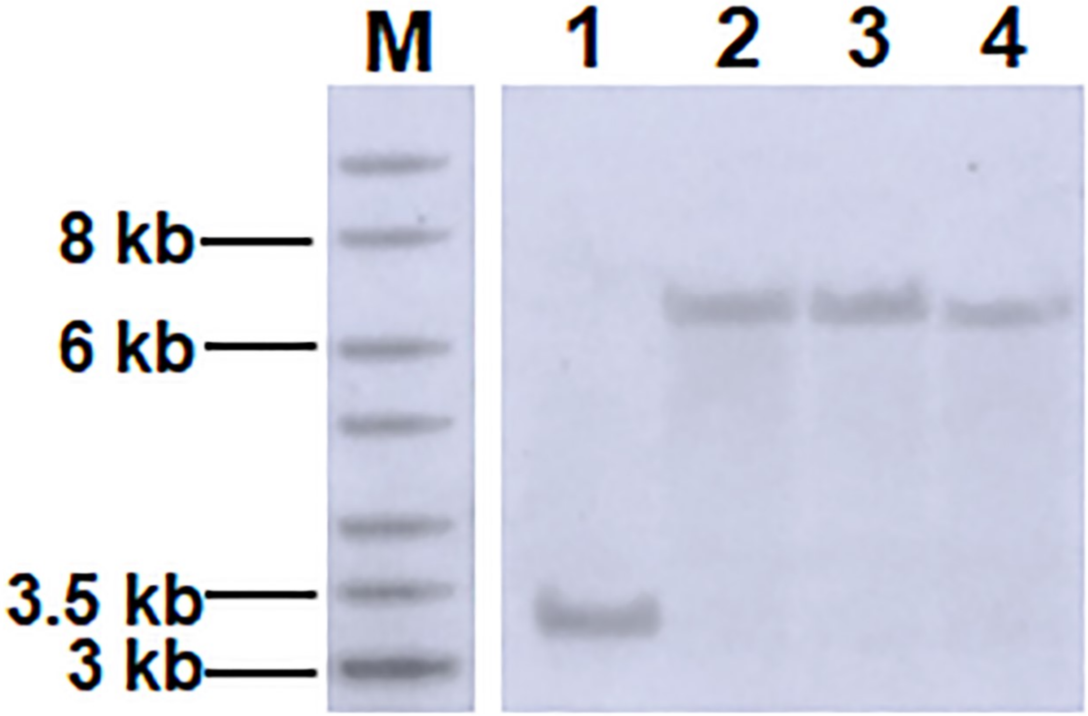

Supplementary Figure 1. Southern hybridization analysis of the deletion mutant Δ*TP05746*. M, 1-kb DNA marker; lane 1, Δ*TpKu70*; lane 2, Δ*TP05746*-8; lane 3, Δ*TP05746*-9; and lane 4, Δ*TP05746*-11.

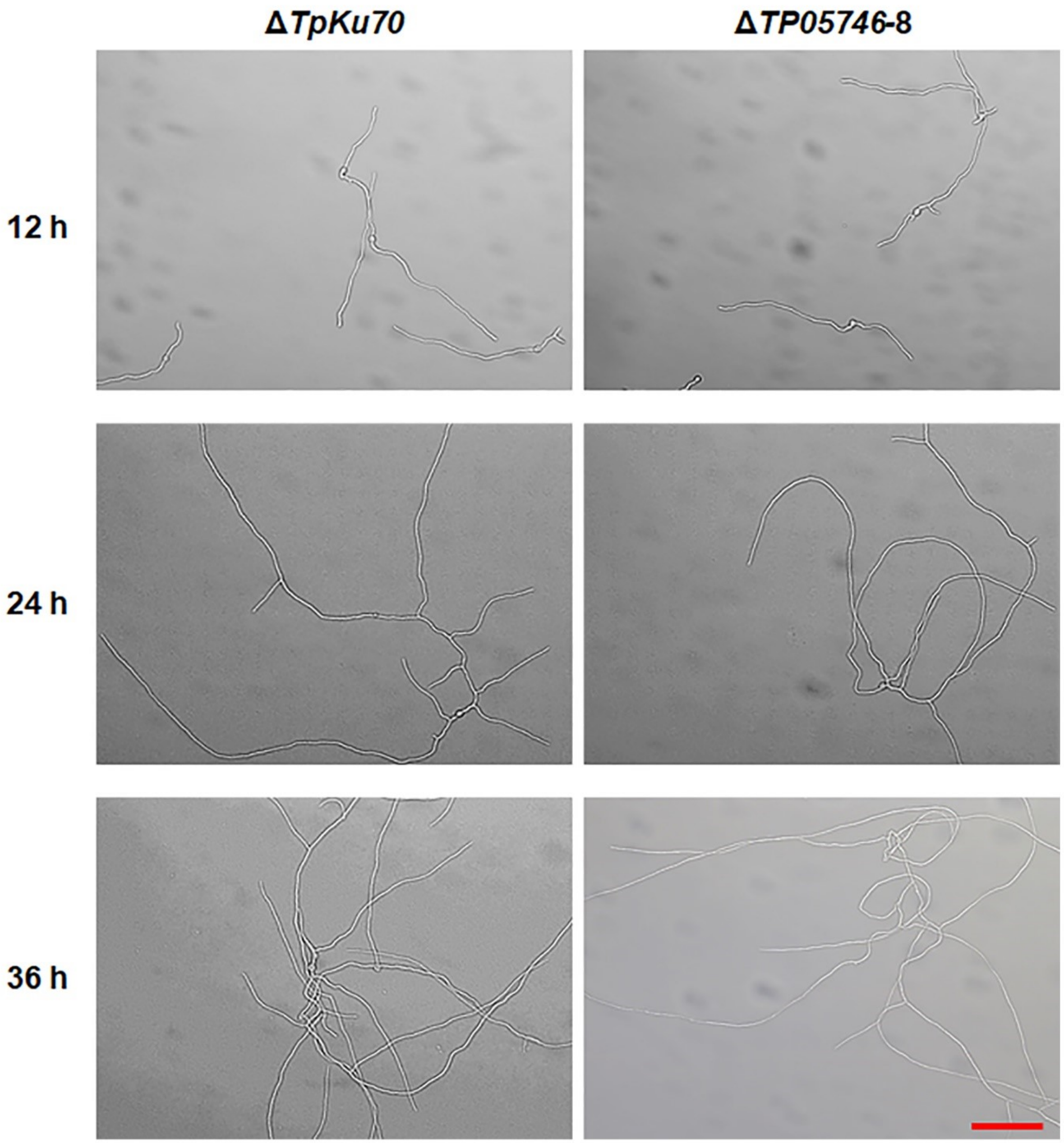

Supplementary Figure S2. Microscopic investigation of mycelial growth and sporulation by *T. pinophilus* mutant Δ*TP05746* and parental strain Δ*TpKu70* on SLM containing glucose. Fungal strains were cultured at 28°C for 12–36 h. Red arrows indicate the developing spores. Bar is 50 µm. SLM: standard liquid medium.

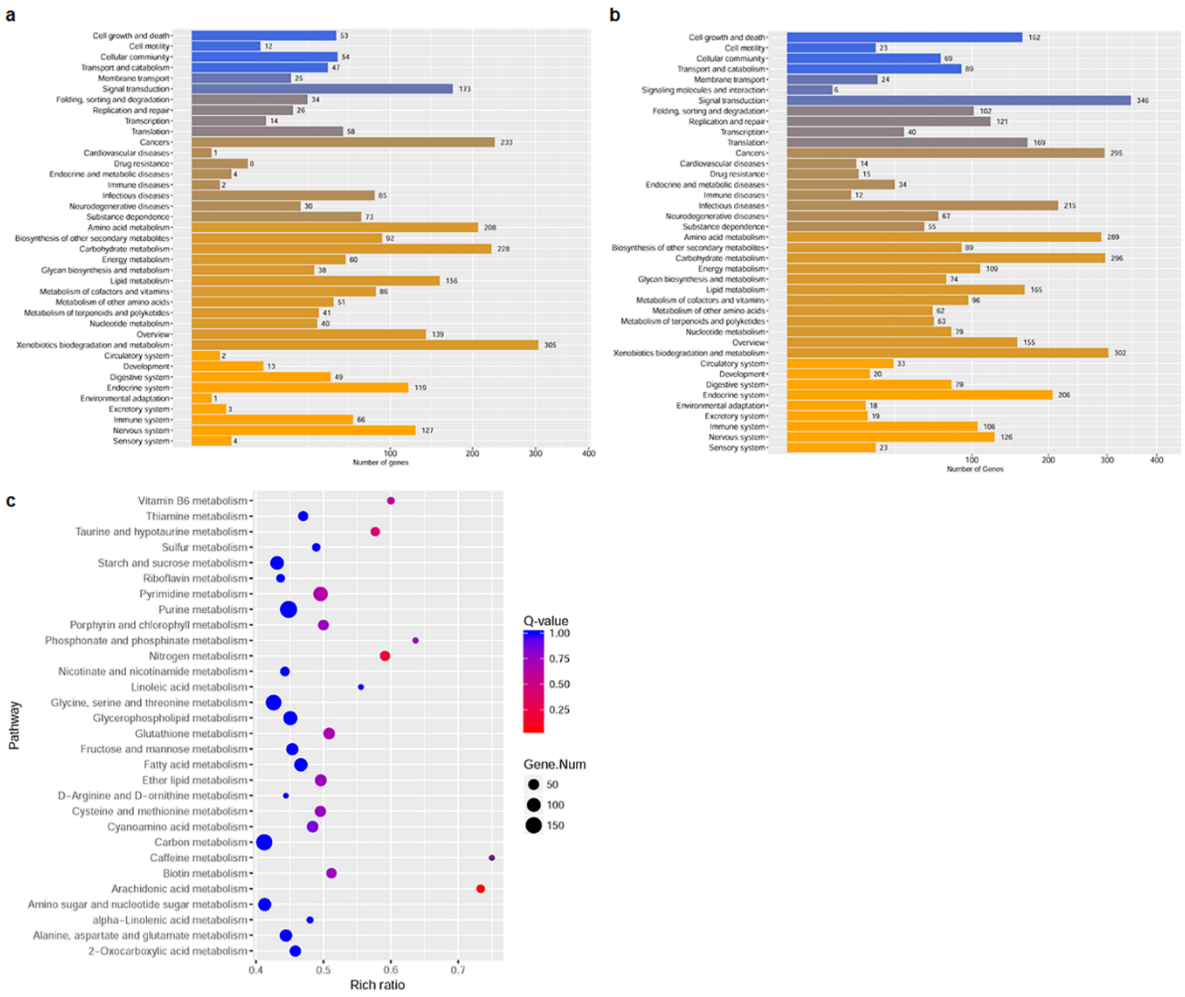

Supplementary Figure S3. KEGG pathway classification annotation of down-regulated DEGs (a) and up-regulated DEGs (b) in the Δ*TP05746*, and the enrichment pathway analysis of DEGs involved in metabolism (c). DEGs: differentially expressed genes; KEGG: Kyoto Encyclopedia of Genes and Genomes.

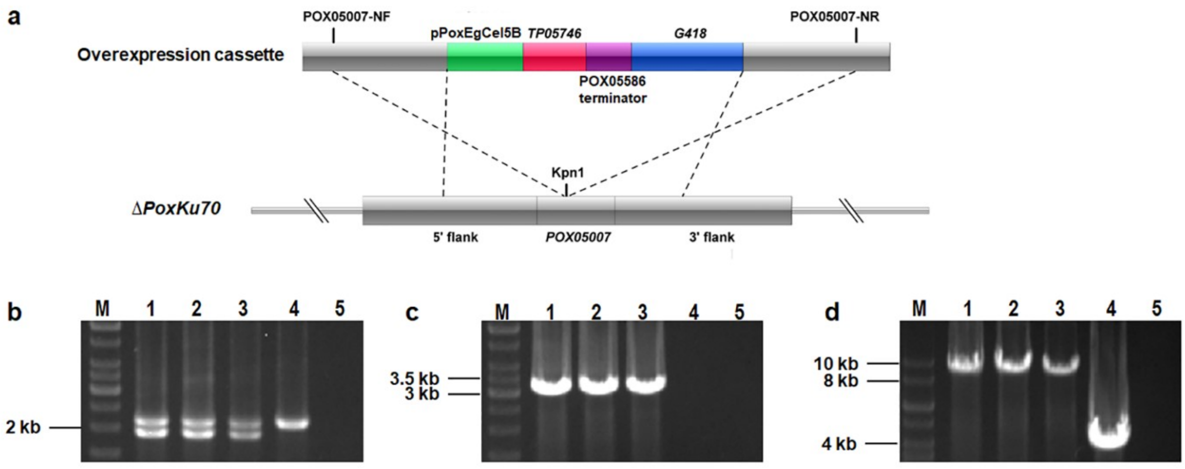

Supplementary Figure S4. Construction of overexpressed strain OX*TP05746_POX*. (a) Schematic illustration. (b-d) PCR confirmation. M, 1 kb DNA markers; Lane 1, OX*TP05746_POX*-4; Lane 2, OX*TP05746_POX*-5; OX*TP05746_POX*-7; Lane 4, Δ*PoxKu70*; Lane 5, ddH_2O. (b) PCR using primer pair G418-F/G418-R; (c) PCR using primer pair POX01166-F/TP05586-R; (d) PCR using primer pair POX05007-NF/POX05007-NR.

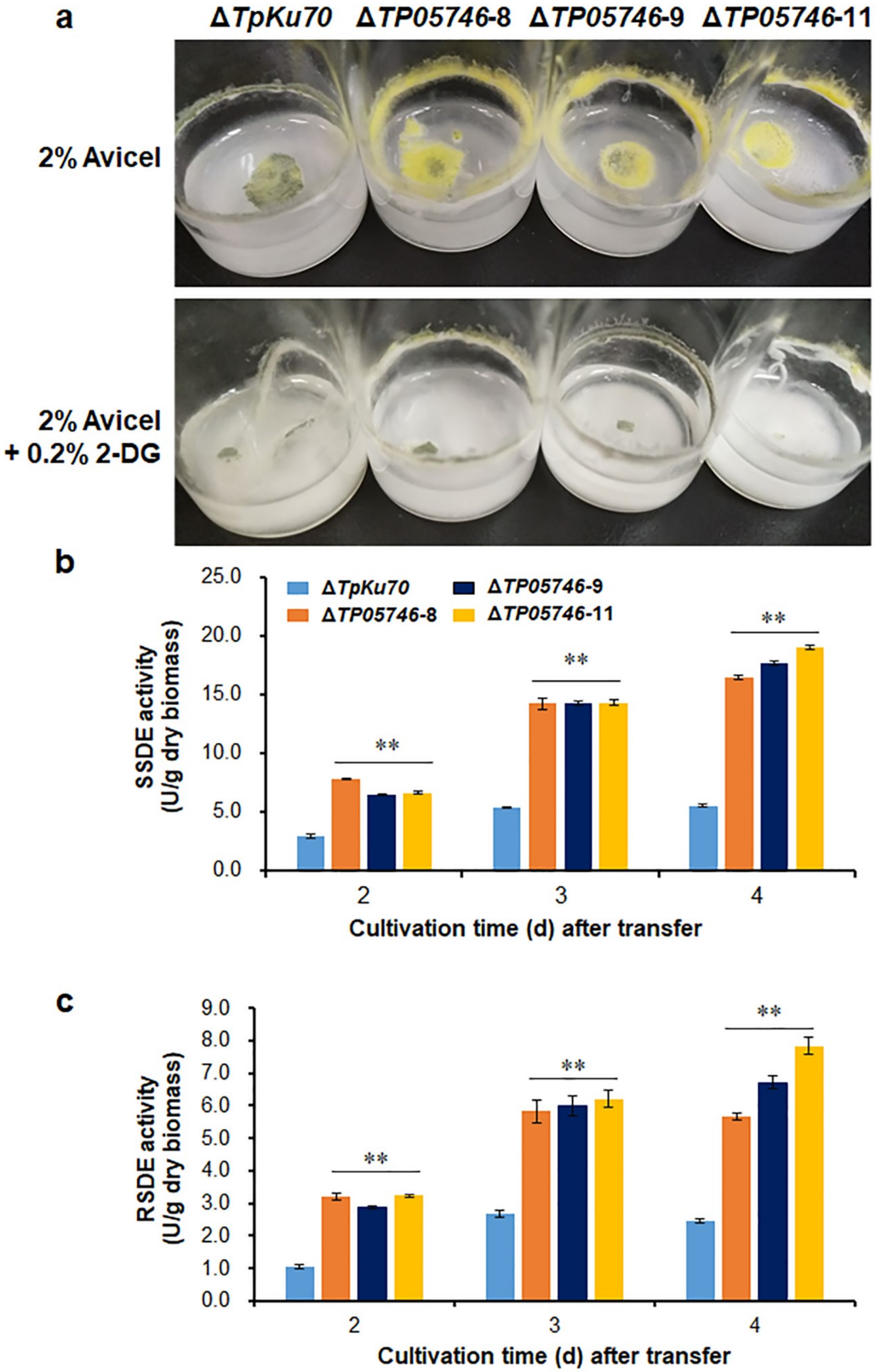
a
ΔTpKu70
ΔTP05746-8
ΔTP05746-9
ΔTP05746-11
2% Avicel
2% Avicel
+ 0.2% 2-DG
b
ΔTpKu70
ΔTP05746-8
ΔTP05746-9
ΔTP05746-11
SSDE activity
(U/g dry biomass)
25.0
20.0
15.0
10.0
5.0
0.0
**
2
3
4
Cultivation time (d) after transfer
c
RSDE activity
(U/g dry biomass)
9.0
8.0
7.0
6.0
5.0
4.0
3.0
2.0
1.0
0.0
**
2
3
4
Cultivation time (d) after transfer

Supplementary Figure S5. Growth of mutant Δ*TP05746* and Δ*TpKu70* in SLM with 2-DG (a) and SSDE (b) and RSDE (c) production of mutant Δ*TP05746* and Δ*TpKu70* in glucose medium. In the a, fungal strains were cultured at 28°C for 5 d, while for 2–4 d in the b & c. ** $p \leq 0.01$ indicates differences between the deletion mutant Δ*TP05746* and the parental strain Δ*TpKu70* by Student's t test. SSDE: soluble-starch-degrading enzyme; RSDE: raw-starch-degrading enzyme; 2-DG: 2-deoxy-glucose.
