# Supplementary material for: Identification of a Novel Transcription Factor TP05746 Involved in Regulating the Production of Plant-Biomass-Degrading Enzymes in Talaromyces pinophilus
Source: Front Microbiol. 2019 Dec 13;10:2875. doi: 10.3389/fmicb.2019.02875 (PMC6923684; doi:10.3389/fmicb.2019.02875)
Supplement: Supplementary file 2 [file Table_1.pdf]

Supplementary Table S1. The primers used in this study.

Primer name	Sequence (5’–3’)
**Primers used for RT-qPCR**	
RT-TP11464-F	AAGGGTTGCGACGGTTTC
RT-TP11464-R	AGGGAGTATCGGGGTTTGTG
RT-TP12265-F	CAAATGCGGTGAGTAGTGCTG
RT-TP12265-R	TCAAACATTCACCTCCCAGC
RT-TP00071-F	CCATACTGGGGTTTCGGTTT
RT-TP00071-R	CGTTGAAGGCAGCATAATCC
RT-TP04013-F	AATGTTCAGACAGGGCACGA
RT-TP04013-R	GACCGATAATAAATGGACGCTT
RT-TP03368-F	GGAATGCGTGGGTCAAGTCA
RT-TP03368-R	CGCCATAATCCAGTTCTCCAATAC
RT-TP07411-F	TGATGGTTGATGTGGTGGCG
RT-TP07411-R	GACTGGTTGAACGGAACAAAGAC
RT-TP12319-F	TCTTTACCATCGCCGTCCAG
RT-TP12319-R	TGAGAAACACAGTCGGGGCA
RT-TP04014-F	GGACAGATTTGCCCGAACAG
RT-TP04014-R	TCCAAACAGCAGTGAATCCCA
RT-TP09781-F	CCGATGAGAACGGAAAAGCG
RT-TP09781-R	TTCAACGATAGCACTGCCCTC
RT-TPactin-F	TCGCTCTTCCTCACGCTATTT
RT-TPactin-F	GATGTCACGGACGATTTCACG
RT- TP08514-F	CGGGGCTCTTTATCTCTCTGA
RT- TP08514-R	GTTGCCTCGCCATTTGCT
RT- TP09412-F	ATAACACCCACTACCAAATCTTCG
RT- TP09412-R	GCACCGTTCAAACCGCAA
RT-TP05820-F	TACGCAATGTCCTCAAACTCG
RT-TP05820-R	CAATAGACTCAGCAGCGGCAC
RT-TP09024-F	GGGGACAATGCGGAGGA
RT-TP09024-R	TTGGCACTGACTGTAGTAAGCG
RT-TP06128-F	CCAGCAAACAGGAGACAATACG
RT-TP06128-R	GCAAGCGTCAAGCGGTGTA
RT-TP09286-F	TCACAGCCACCATCTCAATCC
RT-TP09286-R	TGTGGAGTGAGTTGTTCGGGT
RT-TP00292-F	CGTCGTCGCAAAGTCAAGTG
RT-TP00292-R	AAGTAACCGCTTCGCCCA
RT-TP04237-F	GAACTCCCGACGAAAACACG
RT-TP04237-R	CCTTGTGCGAAGTAGGGGTG

RT-TP04427-F	GAGAAAGAAGGATGGATGGCA
RT-TP04427-R	GGGATAGATTGTGTTGGTGTCG
RT-TP03987-F	CGGGAAGGAAAGAGGATAACC
RT-TP03987-R	CCAGCGTAGCGAAGAGAAAGAA
RT-TP05733-F	CTCCAATCACTCCTAAAAACATCC
RT-TP05733-R	GTCGCTCGCCACCACG
RT-TP10098-F	GCTTTGCGGACCAGTTCG
RT-TP10098-R	TGTATTATCCAGACTGAGTGGTTCC
**Primers used for probes amplification in EMSA**	
TP03368-L-F	CGCAGATTCACATACATACCAGA
TP03368-L-R-EMSA	FAM-GAAGTGAATGCTCGAACTAAACA
TP03368-L-R-Com	GAAGTGAATGCTCGAACTAAACA
TP04014-L-F	CCACATACCCTTATAAAGCGGA
TP04014-L-R-EMSA	FAM-TAAATGATAGAACTGGTTGAGCGT
TP04014-L-R-Com	TAAATGATAGAACTGGTTGAGCGT
TP12319-L-F	CGGATCATCAACCCAAATCA
TP12319-L-R-EMSA	FAM-GCTTGGCTCAGATTCTTGTCG
TP12319-L-R-Com	GCTTGGCTCAGATTCTTGTCG
TP11464-L-F	AGTCAGCCTGTGTTACCCGAT
TP11464-L-R-EMSA	FAM-GCGGTTTACTGGTGGTGAC
TP11464-L-R-Com	GCGGTTTACTGGTGGTGAC
TP00071-L-F	CTAGTACCGCCACCGGAG
TP00071-L-R-EMSA	FAM-GAGATTGACCGGCACCAA
TP00071-L-R-Com	GAGATTGACCGGCACCAA
TP04013-L-F	GCGCTCACTCTATCCGCTTTA
TP04013-L-R-EMSA	FAM-GCTTACAGACCTGAATTGTTGCTC
TP04013-L-R-Com	GCTTACAGACCTGAATTGTTGCTC
TP09412-L-F	GAGATCGAAAGTTTTGCCTTATTAG
TP09412-L-R-EMSA	FAM-TTTGTCGATTGCTTCTGACTGTT
TP09412-L-R-Com	TTTGTCGATTGCTTCTGACTGTT
TP08514-L-F	CGAATGGCAACTCTGGCAC
TP08514-L-R-EMSA	TAM-GTCGCCATGTTAGACATCAGTC
TP08514-L-R-Com	GTCGCCATGTTAGACATCAGTC
TP05820-L-F	GAGCATTTGAAACTCGCAGTG
TP05820-L-R-EMSA	FAM-TCGATATCAAGCACCATGTTGA
TP05820-L-R-Com	TCGATATCAAGCACCATGTTGA
TP09024-L-F	CGTATGATGGAAACATGGCC
TP09024-L-R-EMSA	FAM-TGTCAATGACGTGAAAGATCAACTA
TP09024-L-R-Com	TGTCAATGACGTGAAAGATCAACTA
TP00292-L-F	ACCTTTATAGAAGGAACTGACGGCT

TP00292-L-R-EMSA	GCAATGTTCTGACGGGGCA
TP00292-L-R-Com	GCAATGTTCTGACGGGGCA
TP06128-L-F	GTATCAGTGCTCCCCCCG
TP06128-L-R-EMSA	GAAATGAGGCTCCCAGTGGT
TP06128-L-R-Com	GAAATGAGGCTCCCAGTGGT
TP09286-L-F	AACTGTTCCAACGCAGGGTC
TP09286-L-R-EMSA	TTGCACTTATCGCAGATCTCG
TP09286-L-R-Com	TTGCACTTATCGCAGATCTCG
TP04237-L-F	ACTTGGTCTGAACTGTTGGTGGA
TP04237-L-R-EMSA	TGGCAAATGTGACAGTTGGAAT
TP04237-L-R-Com	TGGCAAATGTGACAGTTGGAAT
TP04427-L-F	ACCTTTTGATGGCGGCGT
TP04427-L-R-EMSA	AAAGGACGCGGGCTAGTCA
TP04427-L-R-Com	AAAGGACGCGGGCTAGTCA
TP05573-L-F	ACATCAGATAAGCTGCGAGTGG
TP05573-L-R-EMSA	CGCTTTCGGTTAGAGGGGA
TP05573-L-R-Com	CGCTTTCGGTTAGAGGGGA
TP10098-L-F	CGCCACACAGACCCATAACT
TP10098-L-R-EMSA	TCTCAGTCATTGAACCCAGGTC
TP10098-L-R-Com	TCTCAGTCATTGAACCCAGGTC
β-tubulin-F	ACCTCACTTGCTCCGCTCTG
β-tubulin-R	FAM-ACAAACTTCATAGATGGAGTGGACA
**Primers used for probe amplification for Southern hybridization**	
TP05746-T-F	TCGCCGATAAGATGGACACT
TP05746-T-R	CAAATACCGAGCAACGAATGA
**Primers used for DNA binding domain-encoding DNA fragment amplification**	
TP05746-F2	TCGGATCCGAATTCGAGCTCATGAGCCTCACGTACTCAC GAT
TP05746-R2	GCGGCCGCAAGCTTGTCGACTTACGCCGACTGAGGATC ATAC
**Primers used for overexpression construction**	
POX05007-NF	ATGGCACATACGCCCCGTTTCAG
POX05007-NR	GCGTAGATAACGATGTCGGTTCAA
POX01166-F	TGAAGCACAACGCTCCCGGTACCCTCTATGGCCTCAACC AATTACA
POX01166-R	AATCGTGAGTACGTGAGGCTCATTTTGACAACTCGAAATC CA
TP05746-F	ATGAGCCTCACGTACTCACGATT
TP05746-R	TAGTTCTTCGTTGCTATCACTCAGTTACGCCGACTGAGGA TCATAC

TP05586-RF	CTGAGTGATAGCAACGAAGAACTA
TP05586-RR	GGTAATCCTTCTTTCTAGATGCCGCCGAAAACATG
G418-F	TCTAGAAAGAAGGATTACC
G418-R	CGATGAAACCGAAGTCGTAGGTACCGTCGACAGAAGATGATATT
